# Significantly high expression of NUP37 leads to poor prognosis of glioma patients by promoting the proliferation of glioma cells

**DOI:** 10.1002/cam4.3954

**Published:** 2021-07-15

**Authors:** Zhendong Liu, Hongbo Wang, Yulong Jia, Jialin Wang, Yanbiao Wang, Lu Bian, Binfeng Liu, Xiaoyu Lian, Bo Zhang, Zhishuai Ren, Wang Zhang, Weiwei Dai, Yanzheng Gao

**Affiliations:** ^1^ Department of Orthopaedic Henan Provincial People's Hospital People's Hospital of Zhengzhou University School of Clinical Medicine Henan University Zhengzhou China; ^2^ Henan University People's Hospital Henan Provincial People's Hospital Zhengzhou China; ^3^ Henan Provincial People's Hospital Cerebrovascular Disease Hospital People's Hospital of Zhengzhou University People's Hospital of Henan University Zhengzhou China; ^4^ Zhengzhou University People's Hospital Henan Provincial People's Hospital Zhengzhou China; ^5^ Department of Neurosurgery of the First Affiliate Hospital of Harbin Medical University Harbin China; ^6^ Xiangya Hospital Central South University Changsha China

**Keywords:** glioma, molecular marker, NUP37, oncogene, prognosis

## Abstract

**Background:**

The carcinogenic effect of NUP37 has been reported recently in a variety of tumors, but its research in the field of glioma has not been paid attention. The main purpose of this study is to reveal the relationship between NUP37 and prognosis or clinical characteristics of glioma patients.

**Methods:**

First, as a retrospective study, this study included thousands of tissue samples based on a variety of public databases and clinicopathological tissues. Second, a series of bioinformatics analysis methods were used to analyze the NUP37 and glioma samples from multiple databases such as the CGGA, TCGA, GEO, HPA, and GEPIA. Third, to analyze the relationship between the expression level of NUP37 in tumor tissues and cells and a variety of clinical prognostic molecular characteristics, whether it can be an independent risk factor leading to poor prognosis in glioma and whether it has clinical diagnostic value; GSEA was used to analyze the cancer‐related signaling pathways that may be activated by high expression of NUP37. Fifth, CMap was used to analyze small molecule drugs that may inhibit NUP37 expression. Finally, the meta‐analysis of thousands of tissue samples from seven datasets and cell proliferation and migration experiments confirmed that NUP37 has a malignant effect on glioma.

**Results:**

NUP37 is highly expressed in glioma patient tissues and glioma cells, significantly correlates with reduced overall survival, and may serve as an independent prognostic factor with some diagnostic value. Silencing NUP37 suppresses malignant biological behaviors of glioma cells. 4 small molecule drugs that had potential targeting inhibitory effects on NUP37 overexpression.

**Conclusions:**

This study demonstrates for the first time a malignant role of NUP37 in glioma and provides a vision to unravel the complex pathological mechanisms of glioma and a potentially valuable biomarker for implementing individualized diagnosis and treatment of glioma.

## Graphical abstract

The present study used a series of analytical methods and confirmed that the NUP37 expression level was elevated in gliomas and led to a poor prognosis of patients. We partially revealed the mechanism of NUP37 in glioma and provided a novel potential diagnostic and therapeutic target.

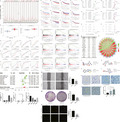

## 1 INTRODUCTION

Gliomas are common malignancies of the central nervous system (CNS) that have poor prognosis. The median survival after standardized treatment for adult glioblastoma multiforme (GBM) is only 14.2 months.^1^ Due to most gliomas grow infiltratively, surgery alone is difficult to remove tumor tissue completely, so it is difficult to achieve ideal therapeutic effect.^2^ Therefore, postoperative radiotherapy and chemotherapy are generally supplemented. However, due to radiation dose limitation and radiotherapy resistance, the survival time of patients was not significantly prolonged. Therefore, many scholars began to pay attention to and apply immunotherapy, molecular‐targeted therapy, alternating electric fields therapy, and so on, but the prognosis of glioma patients has not been greatly improved.^3^, ^4^, ^5^ The reason may be that the complex pathological process of gliomas has not been fully elucidated, and the lack of effective biomarkers for the diagnosis and molecular targeted treatment of gliomas.

Many biological targets are used in the diagnosis and treatment of glioma. For example, EGFR amplification has a high incidence in gliomas, which may be used as a reference index to determine the pathological grades of the tumor.^6^ The Ki‐67 proliferation index is closely connected to tumor differentiation, invasion, and metastasis, which are vital reference indexes for tumor prognosis.^7^, ^8^ PTEN promotes glioma development by interacting with DAXX in glioma.^9^ In addition, molecular markers, such as IDH mutation, TERT, MGTT, VEGF, and TP53, also attracted much attention in gliomas. Although there are many biomarkers for the diagnosis and prognostic evaluation of gliomas, one or several molecular markers are not sufficient because of the complex pathological mechanism and diverse molecular subtypes of gliomas. Therefore, new biomarkers are urgently needed to better understand the pathological process of glioma.

Chromosomal aneuploidy, which is an abnormal chromosome segregation during mitosis, is a common feature and possible cause of cancer. The NUP family, also known as the nuclear pore complex (NPC), occupies a significant position in cell mitosis and kinetochore–microtubule interactions,^10^, ^11^ and it is involved in the cell cycle, apoptosis, and migration.^12^ Its structural and functional abnormalities are associated with a variety of diseases and tumors. For example, aberrant high expression of NUP88 is associated with the occurrence, development, and invasion of rectal tumor and breast cancer, and may be used as a prognostic predictor.^13^, ^14^ NUP98 also plays an indispensable regulatory role in the pathological process of leukemia, and it may become a therapeutic target.^15^, ^16^ The abnormal expression of NUP160‐SLC43A3 promotes the proliferation and development of angiosarcoma cells.^17^ In addition, NUP37 is also concerned with the malignant progression of liver cancer, oral cancer, and non‐small cell lung cancer.^18^, ^19^, ^20^ However, NUP37 as oncogene was not reported in gliomas, especially its relationship with clinical features.

Therefore, the present study examined the relationship between the expression of NUP37 and the clinical features of gliomas through the joint analysis of a large sample size in multiple databases. First, it is reported for the first time that the high expression of NUP37 as an oncogene is obviously associated with the prognosis of glioma especially grades III. Second, by gene set enrichment analysis (GSEA), we identified the possible oncogenic pathway of NUP37. Third, we identified four small molecule compounds that may inhibit the expression of NUP37 using the CMap database. Finally, we verified the effect of NUP37 on the behavior of glioma cell line by traditional experimental methods. Therefore, we suggest that NUP37 is a valuable potential biomarker and molecular target for the diagnosis and treatment of glioma.

## 2 MATERIALS AND METHODS

### 2.1 Data collection

The Chinese Glioma Genome Atlas (CGGA, http://www.cgga.org.cn/) is a genomics database that contains 2000 Chinese glioma specimens. The mRNA‐seq data of 1018 gliomas and microarray data of 301 glioma specimens were obtained from this database, and matching clinical information was acquired. Specimens that lacked information, such as survival time and molecular characteristics, were removed. Finally, 748 cases of mRNA‐seq data and 268 cases of microarray data were retained for subsequent analysis and processing. The basic clinical characteristics of the sample are shown in Tables S1 and S2.

The Cancer Genome Atlas (TCGA, https://portal.gdc.cancer.gov/) is the most widely used public tumor database, and it contains RNA‐seq, miRNA‐seq, methylation data, and matching clinical information for a variety of tumors. The present study obtained 698 cases of glioma mRNA‐seq data from the database. After excluding the specimens that lacked relevant clinical information, 653 specimens were included for follow‐up research. The basic clinical characteristics of the included specimens are shown in Table S3.

Gene expression omnibus (GEO, https://www.ncbi.nlm.nih.gov/geo/) database is an open biological database that contains high‐throughput gene expression data submitted by research institutions around the world. We retrieved two datasets from the database: GSE50161 and GSE116520. GSE50161 contained 34 glioma and 13 normal brain tissue specimens, and the GSE116520 contained 34 glioma and 8 normal brain tissue specimens.

Gene Expression Profiling Interactive Analysis (GEPIA, http://gepia.cancer‐pku.cn/) is an online data analysis and visualization tool that contains the RNA sequencing data of various human tumors and matching normal tissues.^21^ It can analyze the expression level of single gene in different tumors and its effect on prognosis. We obtained the expression data of NUP37 in various tumors and normal tissues from this database for further analysis. It contains 163 high‐grade glioma specimens, 518 low‐grade glioma specimens, and 243 normal brain tissues.

The Human Protein Atlas (HPA) uses transcriptomic and proteomic techniques to study the protein expression in different human tissues and organs at protein levels.^22^ The database can be logged in on this link (https://www.proteinatlas.org/). Three normal brain tissues, two low‐grade gliomas and one high‐grade gliomas were randomly downloaded to detect the protein expression of NUP37 in gliomas and normal brain tissues at all levels were obtained from this database.

### 2.2 Gene set enrichment analysis

Gene set enrichment analysis (GSEA) is a bioinformatics analysis tool that is widely used to annotate and predict gene functions. After normalization of the above three sets of data (CGGA mRNA‐seq, CGGA microarray, and TCGA mRNA‐seq), the specimens were split into high and low expression groups according to the expression level of NUP37. To reveal how NUP37 participated in the pathological process of glioma, we performed enrichment analysis using GSEA 4.0.2 jar software. The number of permutations was set to 1000, and the genome database was set to the KEGG cell signaling pathway. Normal *p* < 0.05 and FDR <0.25 were considered significantly enriched.

### 2.3 Connectivity map analysis

Connectivity Map (**CMap**, https://portals.broadinstitute.org/cmap/) is an online drug analysis tool that discovers and predicts potential therapeutic drugs for certain diseases based on genome expression profiles.^23^ We first used Pearson correlation coefficient to obtain the top 10 positive and negative correlation genes of NUP37 based on the RNA‐seq datasets of 748 glioma samples in CGGA databases. Then, we regard these genes as upregulated or downregulated genes based on the expression relationship with NUP37. Then, we use the GPL570 platform to convert related genes into probe information, and then upload it to the CMap tool to analyze potential inhibitors of NUP37. The negative correlation results (*p* < 0.01 and enrichment < −0.7) were identified as potential inhibitors of NUP37. In addition, we also obtained the 3D chemical structure of these small molecule drugs using PubChem (https://pubchem.ncbi.nlm.nih.gov/).

### 2.4 Cell culture and transfection

Glioma cells (LN229, A172, and T98 cells) and Human astrocytes cells (HA cells) were obtained from the Cell Bank of the Chinese Academy of Sciences. Cells were cultured in DMEM medium with 10% FBS (GIBCO), 100 U/ml penicillin, and 100 mg/ml streptomycin (Invitrogen) at 37°C with 5% CO_2_. The short interfering (si)RNA to NUP37 was purchased from GenePharma. The siRNA sequence was as follows: sense, 5′‐GCAUGUGGUAGAAUUUAAUTT‐3′ and antisense, 5′‐AUUAAAUUCUACCACAUGCTT‐3′. The negative control (NC) siRNA sequence was sense, 5′‐UUCUCCGAACGUGUCACGUTT‐3′ and antisense, 5′‐ACGUGACACGUUCGGAGAATT‐3′. Cells were transfected using Lipofectamine 2000 reagent (Invitrogen; Thermo Fisher Scientific, Inc.) following the manufacturer's protocol. After 24 h of transfection, the knockdown efficiency was detected by RT‐qPCR technology.

### 2.5 Clinical samples

Glioma tissues and normal brain tissues that underwent surgical treatment for glioma or primary epilepsy were obtained from the operating room. A total of 49 glioma tissues and 12 normal brain tissues (epilepsy was treated surgically) were obtained. These tissue samples were further embedded in paraffin to detect the protein expression of NUP37 by immunohistochemistry (4 normal brain tissues and 26 glioma tissues). In addition, the other 8 patients with epilepsy and 23 patients with glioblastoma were collected and stored in liquid nitrogen, and the mRNA level of NUP37 was detected by RT‐qPCR. The diagnosis of glioma was pathologically confirmed. All patients provided written informed consent. The study was approved by the ethics committee.

### 2.6 RT‐qPCR analysis

Total RNA from cells was extracted using the Total RNA kit I (Omega Biotek). RNA concentration was detected using Nanodrop (Thermo Fisher Scientific). RNA was reverse‐transcribed into cDNA by NovoScript Plus All‐in‐one 1st Strand cDNA Synthesis SuperMix (Novoprotein). Finally, RT‐qPCR was performed to detect the expression level of NUP37 using Novostart SYBR qPCR SuperMix Plus Kit (Novoprotein). Primers were manifested as follows: GAPDH forward 5′‐CAAGGTCATCCATGACAACTTTG‐3′ and GAPDH reverse 5′‐GTCCACCAC CCTGTTGCTGTAG‐3′; NUP37 forward 5′‐TAGGACACCCTCAGCCCATC‐3′ and NUP37 reverse 5′‐TTCAGTCACCCAAAACAACA‐3′. The relative NUP37 expression levels were determined using the 2^−ΔΔCT^.

### 2.7 Wound healing assay

A172 cells to make single‐cell suspension, then cells were seeded in 6‐well plate at 2 × 10^5^/well and incubated in an incubator at 37℃ with 5% CO_2_. After the cells had grown to confluence, a linear scratch in the cell monolayer was made with a 200 µl sterile pipette, the microscope was immediately applied for photograph, and the migrated distance was observed after 24 h. The distance migrated by cells in each group was measured using ImageJ software (v.1.52r, National Institutes of Health).

### 2.8 Colony formation analysis

A172 cells were prepared as a single‐cell suspension and then seeded in 6‐well plate at 500 cells/well. After 14 days of culture, the cells were fixed using paraformaldehyde for 20 min and stained with Crystal violet solution (Solarbio). Colony numbers were counted using ImageJ software (v.1.52r, National Institutes of Health).

### 2.9 Immunochemical staining

The 4 normal brain samples, 7 low‐grade gliomas samples, and 19 high‐grade gliomas samples were collected from the operating room for NUP37 immunochemical stabilization. For immunohistochemical (IHC) staining, tissue sections of 2 µm thickness were placed in xylene and graded alcohols for deparaffinization and hydration. Antigen retrieval was performed using a microwave oven in EDTA (pH 8.0) buffer for 15 min. Blocking was performed with 10% goat serum. The appropriate amount of primary antibody NUP37 (1:100, Bioss) working solution was then dropped on the sections and incubated overnight at 4℃. The staining results were observed under light microscope and photographed. IHC results were analyzed using Image‐Pro Plus software (version 6.0).

### 2.10 Immunofluorescence staining

The treated cells were fixed with 4% paraformaldehyde for 30 min and then perforated with 0.1% Triton X‐100 for 10 min at room temperature. Primary antibody Ki67 (1:100, Abbkine) was added and incubated overnight at 4℃. Then, incubated with DyLight 594‐conjugated AffiniPure goat anti‐rabbit IgG (1:500 dilution; Boster Biological, BA1142) in a humidified atmosphere at room temperature in the dark for 1 h. Finally, the staining results were observed and photographed under a fluorescence microscope.

### 2.11 Meta‐analysis

In order to find more evidence to confirm the effect of NUP37 on the prognosis of glioma, we used meta‐analysis to test that NUP37 is a risk factor for the prognosis of glioma patients. First of all, we searched several databases (The PubMed; Web of Science; Embase databases) to find the research about NUP37 in gliomas. Unfortunately, up to now, there is no literature about the relationship between NUP37 and gliomas. Therefore, this study can only use the existing data in the database for meta‐analysis and evaluation of NUP37 on the overall survival of gliomas patients. In this study, we collected seven sets of data (TCGA:698 patients; CGGA:1016 patients; GSE43378:50 patients; GSE4412:85 patients; GSE74187:60 patients; GSE83300:50 patients) including 1959 glioma samples. HR value and 95% CI were considered as an important indicator to evaluate the analysis results. The heterogeneity between multiple datasets was analyzed by the Q test (I^2^ statistics). A fixed effects model or a random effects model was selected for meta‐analysis based on the cut‐off criterion was (I^2^ = 50%) in R 3.4 software.

### 2.12 Statistical analysis

Data were analyzed using the R software (v.3.6.1). NUP37 expression in glioma and normal brain tissues was detected using the Wilcoxon method. The overall survival of the NUP37 high expression and low expression groups was determined using Cox regression and Kaplan–Meier analyses. To analyze the main factors affecting the prognosis of patients, univariate and multivariate Cox analyses were used. The ROC method was used to detect whether NUP37 may be used as an independent prognostic factor for glioma. The relationship between NUP37 expression and the clinical features of the specimens was tested using the Wilcoxon or Kruskal–Wallis test. Finally, the cor.test was used to analyze the correlation between NUP37 and other genes.

## 3 RESULTS

### 3.1 NUP37 is aberrantly expressed in various tumors including gliomas

**FIGURE 1:**
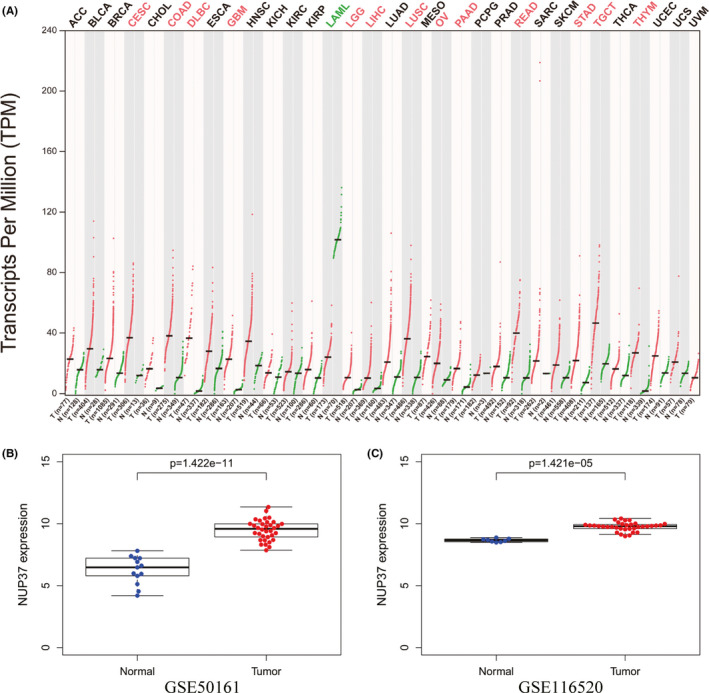
The expression level changes of NUP37 in tumor tissue was compared with that in the control group. (A) GEPIA database showed the differential expression of NUP37 in human tumor tissues (red: NUP37 was highly expressed in tumor tissue; green: NUP37 was low expressed in tumor tissue; black: no statistical significance); (B–C) The expression level of *NUP37* in gliomas was higher than that in the control group in GSE50161 (34 glioma and 13 brain tissue specimens) and in GSE116520 (34 glioma and 8 brain tissue specimens)

Using the GEPIA database, we obtained the expression of NUP37 in different tumors and corresponding normal tissues. As shown in Figure 1A, NUP37 was aberrantly expressed in a variety of tumors. NUP37 was aberrantly highly expressed in glioblastoma multiforme (GBM), brain lower grade glioma (LGG), cervical squamous cell carcinoma and endocervical adenocarcinoma (CESC), colon adenocarcinoma (COAD), lymphoid neoplasm diffuse large B‐cell Lymphoma (DLBC), liver hepatocellular carcinoma (LIHC), and other tumors compared to normal tissues. It was aberrantly lowly expressed in acute myeloid leukemia (LAML). It was worth emphasizing that GEPIA database contains 243 normal brain tissues from the genotype‐tissue expression (GTEx) project and 163 high‐grade gliomas and 518 low‐grade gliomas specimens from TCGA database. Therefore, this database can make up for the deficiency of TCGA database, which cannot analyze the differential expression of genes without normal brain tissue. To deeply examine the role of NUP37 in the pathological process of glioma, we performed further investigations. Using the GEO database, we obtained two datasets of glioma samples and normal tissues for analyses. As shown in Figure 1B,C, the expression of NUP37 was different in these two datasets, and the expression level in glioma specimens was higher than normal brain tissues. Through the analysis of the above databases, NUP37 showed abnormally high expression in glioma. However, the relationship between the significantly high expression of NUP37 and the prognosis of glioma patients is not clear.

### 3.2 Glioma patients with aberrantly high NUP37 expression may have a poor prognosis

**FIGURE 2:**
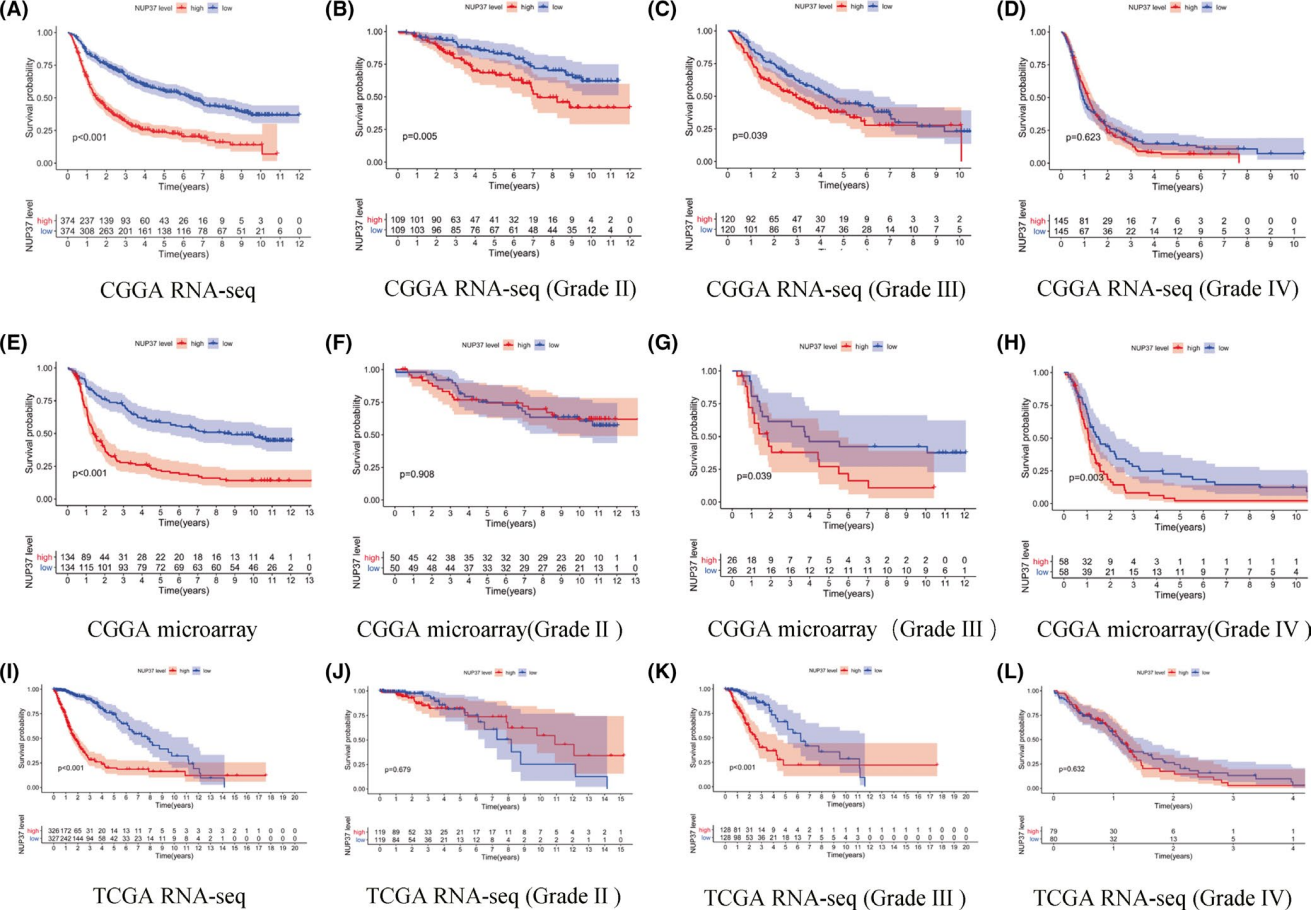
The effect of *NUP37* expression level on overall survival of glioma patients at all levels was based on three different databases (CGGA RNA‐seq; CGGA RNA‐microarray; and TCGA RNA‐seq). The effect of *NUP37* on the overall survival of glioma at all grades (A, E, and I); the effect of *NUP37* on the overall survival of glioma at grade II (B, F, and J); the effect of *NUP37* on the overall survival of glioma at grade III (C, G, and K); the effect of *NUP37* on the overall survival of glioma at grade IV (D, H, and L)

To reveal the role of NUP37 in the pathological process of glioma, we performed a multilevel analysis from glioma data derived from multiple database sources (CGGA RNA‐seq, CGGA microarray, and TCGA RNA‐seq). The above data were divided into two groups according to the expression level of NUP37: high expression and low expression. Survival analysis was performed using the Kaplan–Meier analyses, and the results are shown in Figure 2. From Figure 2A,E,I, among all glioma samples (grade II, III, and IV) patients in the high expression group of NUP37 had significantly worse overall survival than those in the low expression group. To clarify the relationship between NUP37 and patient prognosis in different grades of glioma, we divided the samples into grade II, III, and IV based on the clinical information, and a survival analysis was performed separately. As shown in Figure 2C,G,K, the prognosis of the high expression group of NUP37 was apparently worse than the low expression group in grade III glioma. But there were no completely consistent results among the three datasets in grade II and grade IV gliomas. NUP37 for glioma patients (grades II) suggested a significant adverse effect on the prognosis of patient only in the CGGA RNA‐seq dataset (Figure 2B), but no statistical significance was evident in the remaining two datasets (Figure 2F,J). The data set of CGGA microarray suggested that high expression of NUP37 could significantly reduce the overall survival time of patients (grade IV, [Figure 2H]), but the results did not meet the statistical principle in the remaining two datasets (Figure 2D,L). It is well known that glioblastoma (grade IV) was the most important factors for the impact of prognosis of glioma patient due to highly aggressive growth. But there were not totally consistent results among my three datasets. Therefore, to remedy such a flaw, we collected tissue samples from 23 glioblastoma cases and corresponding clinical information to further explain the influence of the NUP37 on glioblastoma prognosis. This result found that high expression of NUP37 can lead to poor prognosis of glioblastoma patients with statistical significance (Figure S1). However, whether it is an independent prognostic factor must be elucidated.

### 3.3 Aberrantly high expression of NUP37 is an independent prognostic factor in glioma patients

**FIGURE 3:**
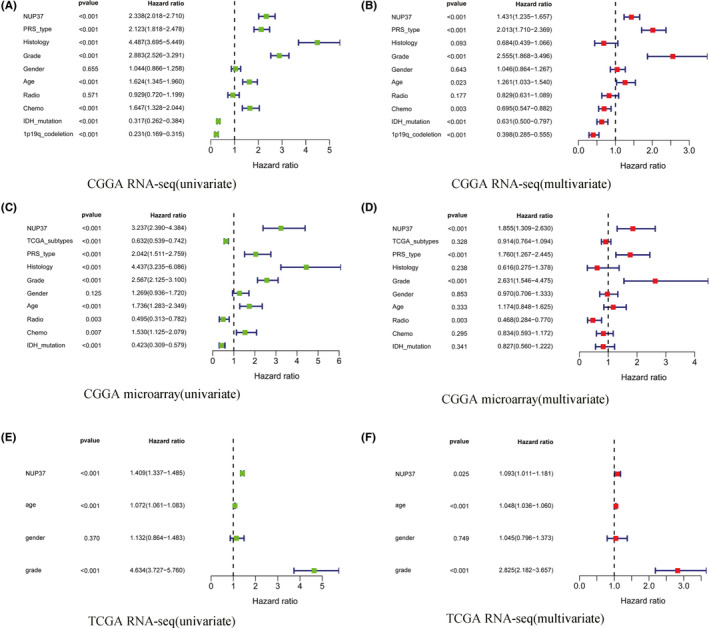
Univariate and multivariate regression analysis of prognosis in glioma patients based on three different databases (CGGA RNA‐seq; CGGA RNA‐microarray; and TCGA RNA‐seq). Univariate analysis of *NUP37* on the prognosis of glioma (A, C, and E); multivariate analysis of *NUP37* on the prognosis of glioma (B, D, and F)

First, we employed univariate analysis of data from the three datasets (CGGA RNA‐seq, CGGA microarray, and TCGA RNA‐seq) to interpret the impact of NUP37 and each clinical feature of the patients on the prognosis of glioma (Figure 3A,C,E).We found that NUP37 was a risk factor for the prognosis in all three databases, and that both tumor grade and the age of the patients were also a risk factor for the prognosis of glioma (*p* < 0.05, [HR] >1). In addition, PRS type, histology, and chemotherapy in databases other than TCGA RNA‐seq also suggest risk for the prognosis of glioma (*p* < 0.05, [HR] >1). Besides, in the CGGA RNA‐seq and CGGA microarray datasets, IDH mutation status was a protective factor for the prognosis of glioma (*p* < 0.05, [HR] <1). Finally, in the CGGA RNA‐seq datasets, 1p19q codeletion status was a low‐risk factor for the prognosis of glioma patients (*p* < 0.001, [HR] = 0.231 (95% CI [0.169–0.315])).

Second, through univariate analysis, we learned that NUP37 or some patient's clinical characteristics were a risk factor for glioma prognosis, but to clarify whether such a result was an independent factor, we adopted multivariate analysis to explain (Figure 3B,D,F). We found that the increased expression level of NUP37 was not affected by other clinical features of glioma patients and was independently a poor prognostic factor for glioma patients in the three datasets (*p* < 0.05, [HR] >1). In addition, there are also some clinical characteristics of patients that was also an independent risk factor for the prognosis of glioma such as: tumor grade, PRS type, histology, and age in some datasets (*p* < 0.05, [HR] >1). Finally, in some datasets such as CGGA RNA‐seq, IDH mutation status, 1p19q codeletion status, and chemotherapy was an independent protective factor for glioma prognosis (*p* < 0.05, [HR] <1). We do not make excessive interpretations of the remaining data, and details are shown in Figure 3B,D,F.

The results of the univariate and multivariate analyses suggest that NUP37 may be used as a prognostic and diagnostic factor for glioma patients, but its diagnostic value must be further examined.

### 3.4 The high expression of NUP37 has a certain diagnostic value for the prognosis of glioma patients

**FIGURE 4:**
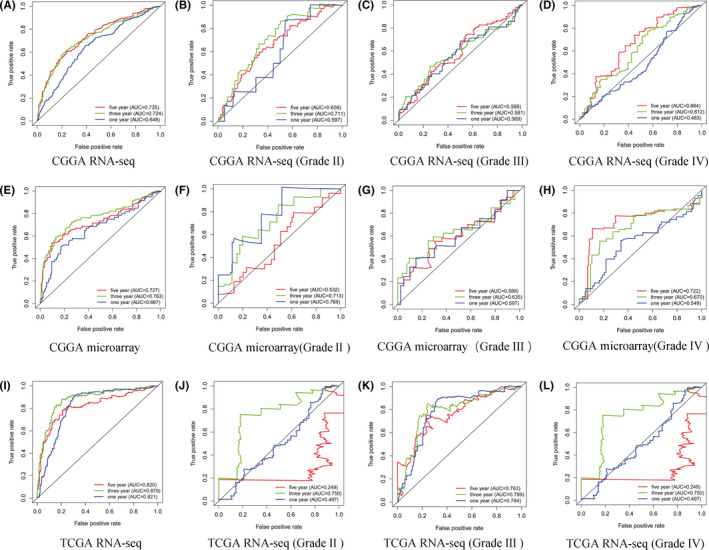
Diagnostic value of *NUP37* expression for poor prognosis in glioma patients based on three different databases (CGGA RNA‐seq; CGGA RNA‐microarray; and TCGA RNA‐seq). The diagnostic value of *NUP37* in all grades of gliomas (A, E, and I); the diagnostic value of *NUP37* in grade II (B, F, and J); the diagnostic value of *NUP37* in grade III (C, G, and K); the diagnostic value of *NUP37* in grade IV (D, H, and L)

ROC curves were used to determine the diagnostic value of high NUP37 expression for glioma prognosis based on the three datasets. As shown in Figure 4A,E,I, the high expression of NUP37 in the three datasets had a better diagnostic value for the 3‐year and 5‐year survival of glioma patients (AUC >0.7). To clarify the diagnostic value of NUP37 for the prognosis in different grades, we divided the samples into grade II, III, and IV on the basis of the WHO classification of clinical information of the samples and plotted ROC curves. As shown in Figure 4K, the high expression of NUP37 in grade III of TCGA RNA‐seq had better diagnostic value for the 1‐year, 3‐year, and 5‐year survival of glioma patients (AUC >0.7). NUP37 also had better diagnostic value for the 3‐year survival of glioma patients of different grades in the three datasets. Overall, the high expression of NUP37 has a certain diagnostic value for the prognosis of glioma patients.

### 3.5 The expression level of NUP37 is associated with multifarious clinicopathological features of glioma

To examine the association of NUP37 with various clinicopathological features of glioma, we analyzed the expression levels of NUP37 in each clinical feature group using the Wilcoxon or Kruskal–Wallis test. As shown in Figure S2A, the expression level of NUP37 increased with WHO grade in the three datasets (*p* < 0.001). As shown in Figure S2D,E, the expression level of NUP37 in the CGGA RNA‐seq and CGGA microarray datasets was significantly reduced in IDH mutations and untreated chemotherapy groups compared to IDH wild and chemotherapy‐treated groups (*p* < 0.001). As shown in Figure S2B,F, NUP37 expression was also clearly correlated with PRS type and 1p19q codeletion status (*p* < 0.001). The expression level of NUP37 was positively correlated with the age of glioma patients (Figure S2C). Figure S2G shows that the expression level of NUP37 was higher in the GBM and recurrent GBM groups. It is not difficult to see through the above data that there is an expression relationship between the expression level of NUP37 and clinical features associated with the prognosis of gliomas, which can also be indirectly inferred that NUP37 was an oncogene in gliomas.

### 3.6 GSEA reveals the cell signaling pathways of NUP37

**FIGURE 5:**
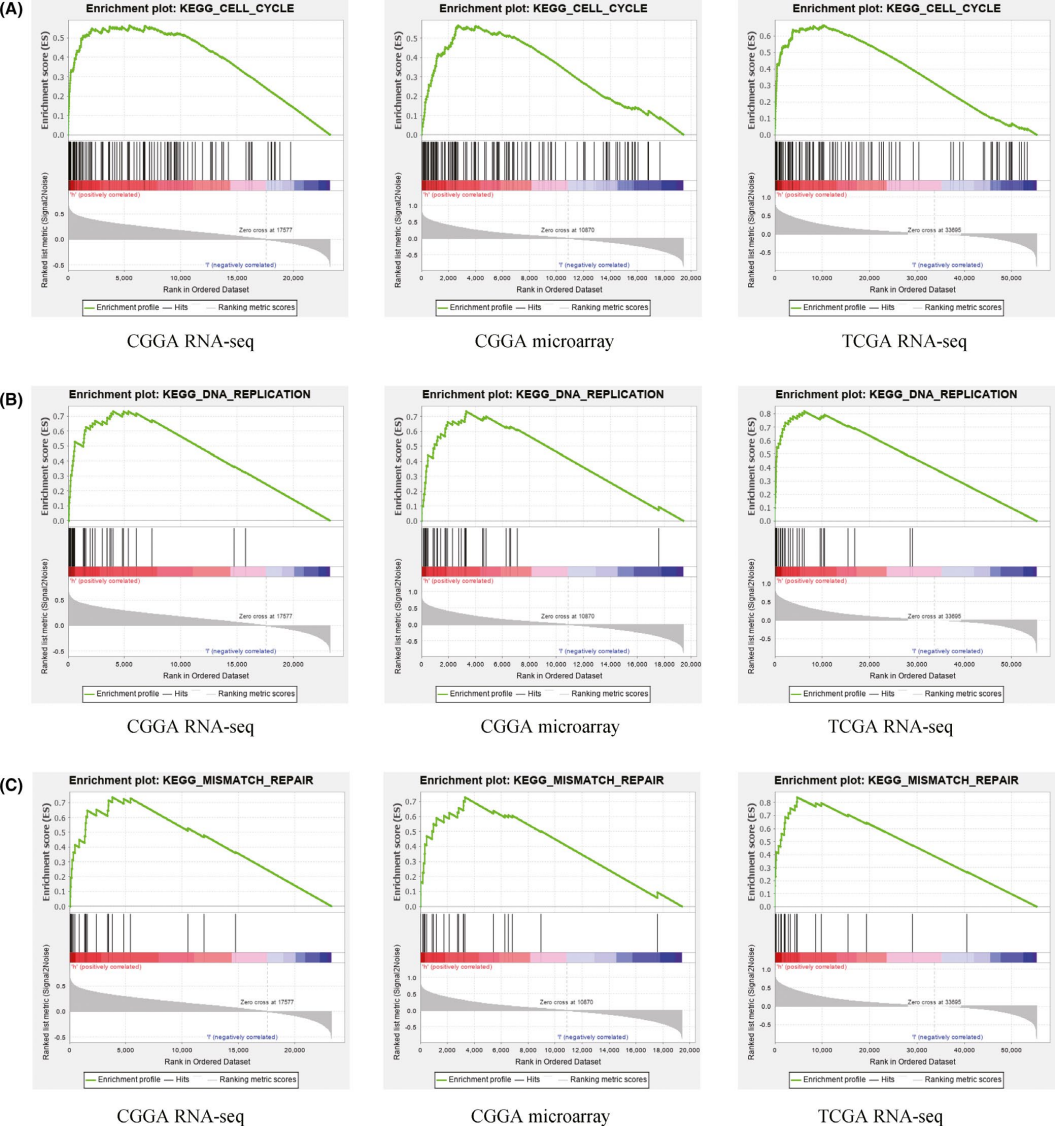
Functional enrichment analysis of *NUP37* in gliomas by GSEA based on three different databases (CGGA RNA‐seq; CGGA RNA‐microarray; and TCGA RNA‐seq). (A) cell cycle, (B) DNA replication, (C) mismatch repair, and (D) homologous recommendation

To indirectly reveal how NUP37 was involved in the pathogenesis of gliomas, we divided the samples into high and low expression groups according to the expression of NUP37 and performed GSEA analysis. As shown in Table 1 and Figure 5, pathways such as cell cycle, DNA replication, mismatch repair, and homologous recommendation, were significantly enriched in the NUP37 high expression group. These results show that NUP37 may participate in the occurrence and development of glioma through these pathways.

**TABLE 1:** Cell signaling pathway that *NUP37* may be enriched

Gene set name	CGGA RNA‐seq			CGGA microarray			TCGA RNA‐seq		
	NES	NOM *p* val	FDR *q* val	NES	NOM *p* val	FDR *q* val	NES	NOM *p* val	FDR *q* val
Cell cycle	1.667	0.03	0.196	1.755	0.021	0.076	2.065	0	0.006
DNA replication	1.76	0.008	0.143	1.727	0.013	0.084	1.912	0	0.016
Mismatch repair	1.815	0.004	0.179	1.758	0.013	0.081	1.995	0	0.012
Homologous recombination	1.71	0.008	0.163	1.808	0.004	0.058	1.979	0	0.008

Note

Gene sets with NOM *p* value < 0.05 and FDR *q* value < 0.25 were considered as significantly enriched.

Abbreviations: FDR, false discovery rate; NES, normalized enrichment score; NOM, nominal.

### 3.7 Co‐expression analysis of NUP37 and drug screening of CMap analysis

**FIGURE 6:**
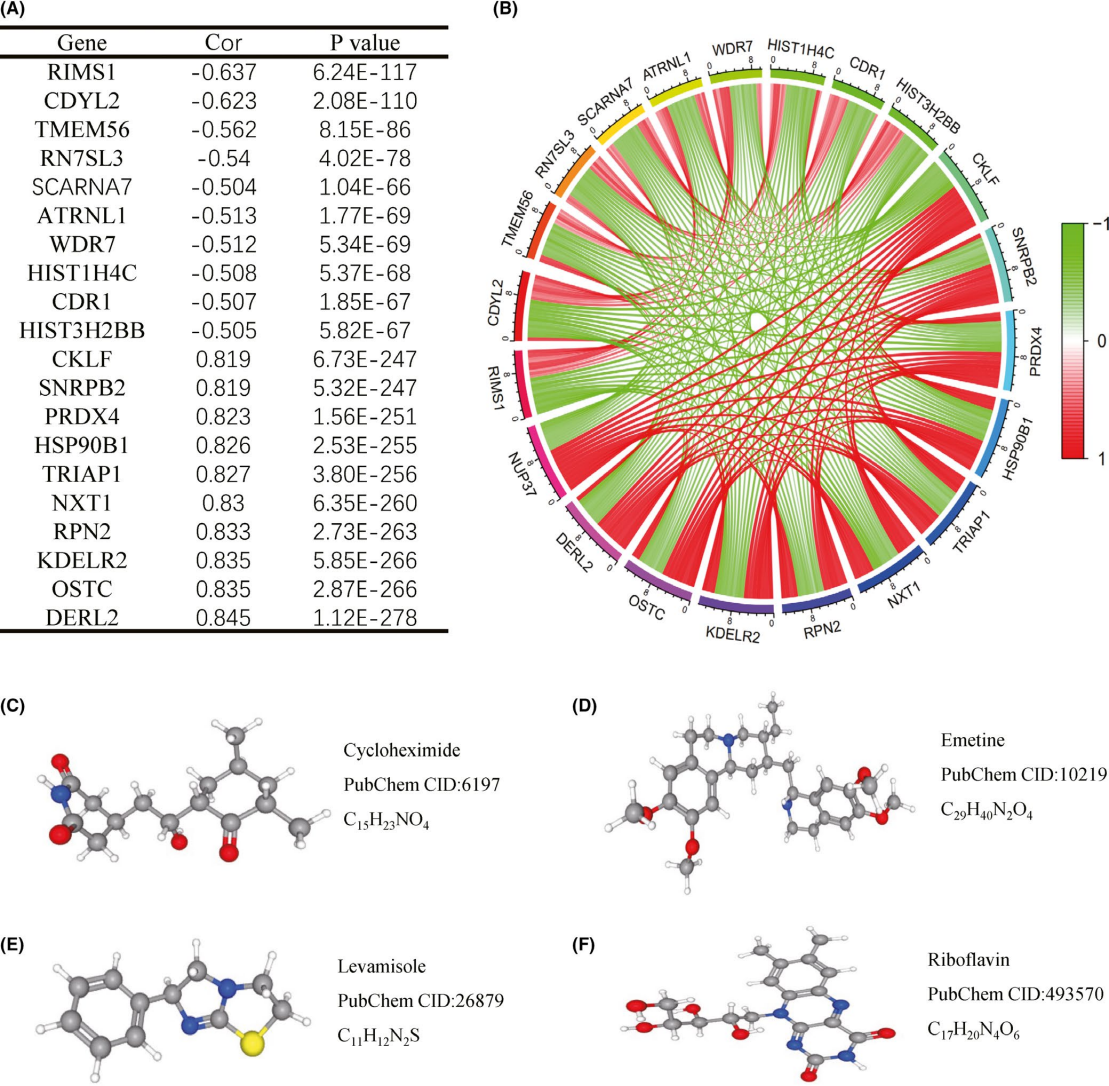
Co‐expression analysis of NUP37 and screening of inhibitors. (A–B) the top 10 genes with positive and negative co‐expression with NUP37 (cor >0 was positively correlated and cor <0 was negatively correlated); (C–F) 3D structure and molecular formula of drug with inhibition of NUP37 expression (C: cycloheximide, D: emetine, E: levamisole, and F: riboflavin)

In order to understand the expression relationship between NUP37 and other genes, we employed Pearson's correlation coefficient to explore this part based on the dataset of CGGA RNA‐seq. Subsequently, we found that hundreds of genes have a co‐expression relationship with NUP37, and further we show the top 10 genes, CKLF as a representative, most positively correlated with NUP37, and the most negatively correlated genes, RIMS1 as a representative, in Figure 6A,B. The theoretical basis of gene co‐expression infers the function of unknown genes through known gene dereplication, therefore, this part of the study will help to reveal NUP37 function in glioma. In order to obtain for drugs with inhibitory effects on NUP37, CMap assays were employed. Based on the rationale of CMap analysis, we loaded the genes with positive correlation to NUP37 as upregulated genes and the genes with negative correlation as downregulated genes into the CMap database to obtain drugs with inhibitory effect on NUP37. Four small molecule drugs were screened according to this criteria, and the results are shown in Table 2. The 3D structures and specific information of these drugs are shown in Figure 6C–F.

**TABLE 2:** Results of cMap analysis

Rank	cMap name	Enrichment	*p* value
1	Cycloheximide	−0.802	0.00296
2	Riboflavin	−0.8	0.00316
3	Levamisole	−0.758	0.00696
4	Emetine	−0.714	0.01343

Abbreviation: CMap, connectivity map.

### 3.8 Meta‐analysis of NUP37 expression and relationship between known biomarkers in glioma

**FIGURE 7:**
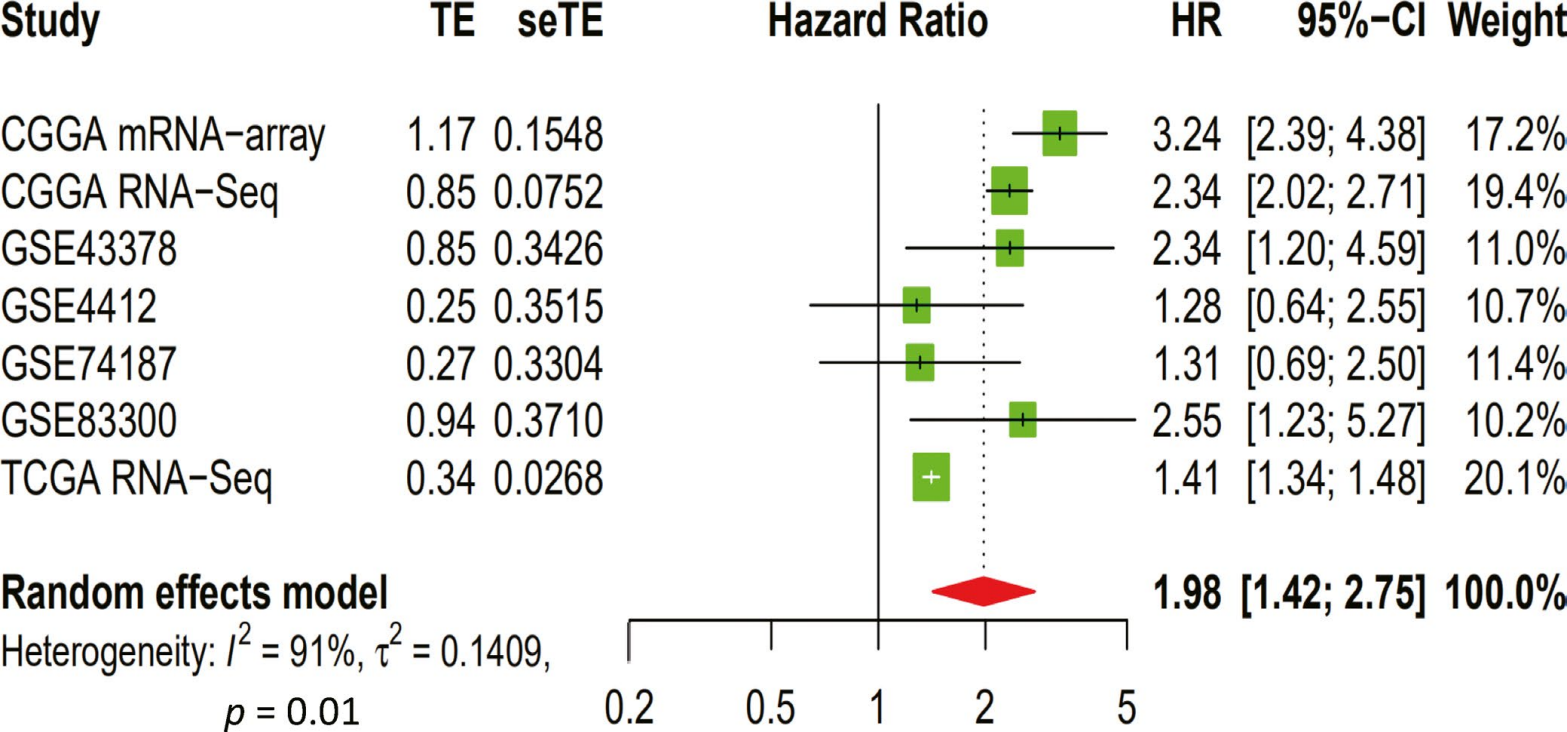
One risk factor for the prognosis of gliomas was the overexpression of NUP37 in Forest plot from seven different datasets

We retrieved multiple well‐known databases and found no reports about NUP37 in glioma, so we could only collect the extant data in the database to complete the meta‐analysis to further confirm the effect of NUP37 on the prognosis of glioma patients. A total of 7 datasets containing 1959 glioma samples were included in this analysis, and the results of the meta‐analysis found that each individual dataset revealed NUP37 as a risk factor for glioma prognosis ([HR] >1). The results of the seven datasets as a whole showed that NUP37 was also a risk factor for glioma prognosis ([HR] = 1.98 (95% CI [1.42–2.75])). Because the heterogeneity among the seven datasets was greater than the cut‐off criterion was (I^2^ = 50%), we could only choose a random effects model to present the results in Figure 7.

Although there is so much evidence that NUP37 was a risk factor for the prognosis of glioma, we try to further explore the relationship between NUP37 and known biomarkers in glioma, which provide more evidence to support NUP37 as a malignant molecule. Hence, we use the method of co‐expression to explore the relationship between them based on the dataset of CGGA RNA‐seq. The results showed that there was a positive correlation between NUP37 and most known biomarkers of glioma such as ATRX, EGFR MGMT, CDK4, and so on (Figure S3A–N).

But only BRAF was negatively correlated with the expression of NUP37 (Figure S3O). Although the above results help to improve the understanding of the impact of NUP37 on the prognosis of glioma, these evidences were indirect and more effective experimental evidences need to be provided.

### 3.9 Verification of the reliability of NUP37 analysis in glioma

**FIGURE 8:**
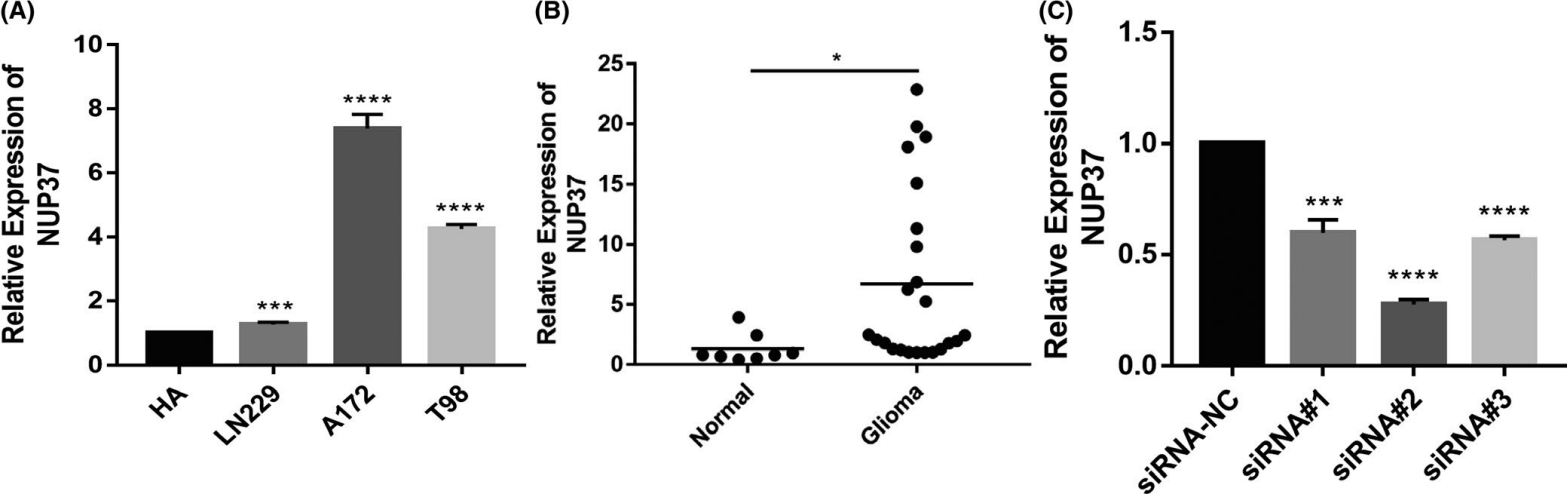
High expression of NUP37 in glioma tissues and cell lines. The difference of NUP37 expression between glioma cell lines and astrocytes was detected by RT‐qPCR (A); the expression of NUP37 was detected by RT‐qPCR between glioma tissues and normal brain tissues (B); the interference efficiency of NUP37 was detected by RT‐qPCR after transfection of the NUP37 knockdown in the A172 cell lines (C). Data are mean ± SD, *p < 0.05, **p < 0.01, ***p < 0.001, and ***p < 0.0001

**FIGURE 9:**
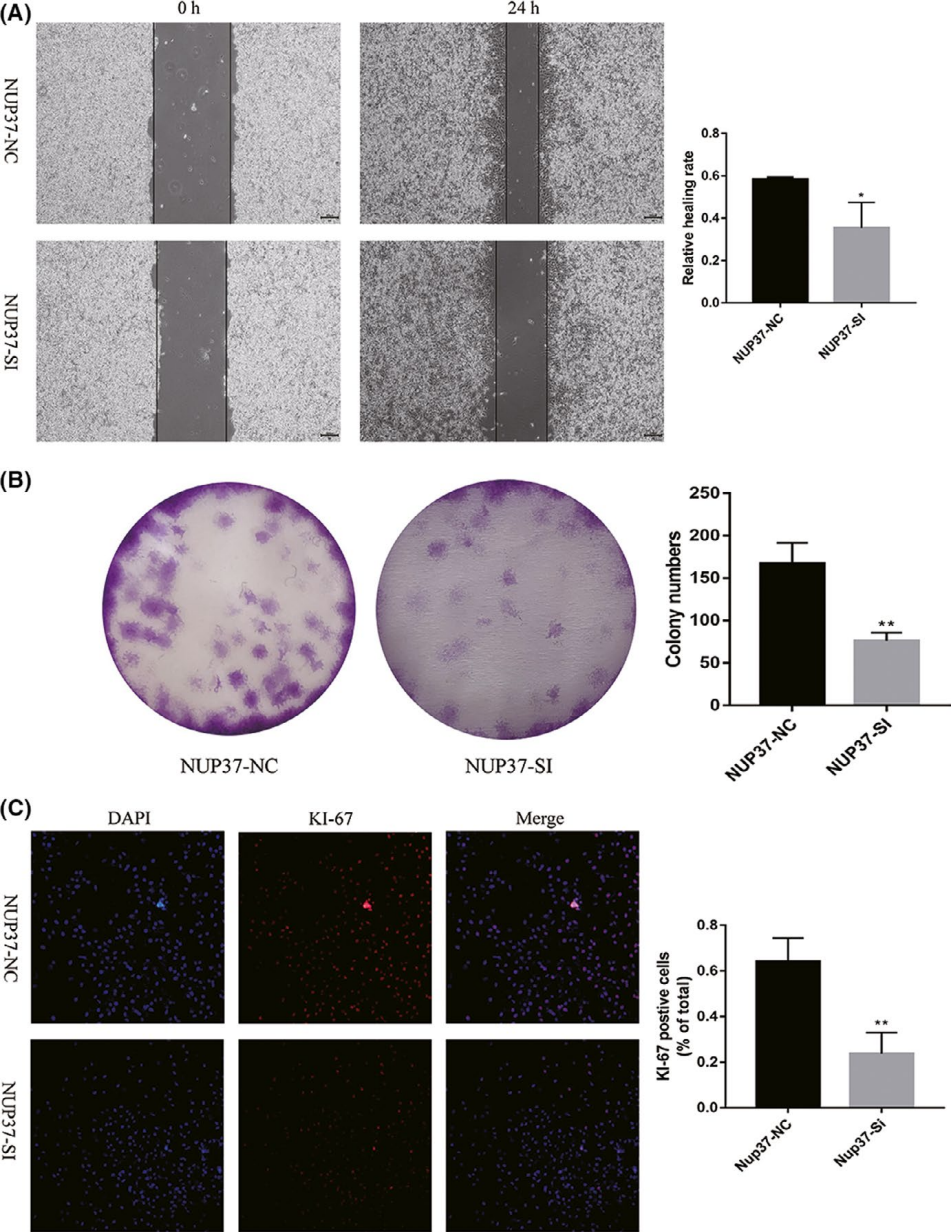
The proliferation and migration of A172 glioma cell line were inhibited by knockout of NUP37. Cell wound healing assay was used to detect the crawling ability of A172 cells after knockout of NUP37 (A), magnification: 20×. Clone formation assay and ki67 staining were used to verify the effect of NUP37 knockout on the proliferation of A172 cells (B). Data are mean ± SD, *p < 0.05, **p < 0.01. The expression of NUP37 was correlated with the progression of the tumorigenesis of glioma

The above database analysis results have fully revealed that the increased expression of NUP37 was an independent risk factor leading to the decrease of overall survival time of patients with gliomas. However, in order to demonstrate the role of NUP37 in glioma more scientifically and rigorously, it is necessary to use traditional experimental methods to verify the analysis results. First, we used RT‐qPCR to examine the expression levels of NUP37 in glioma cell lines and tissues. As shown in Figure 8A,B, the expression level of NUP37 was obviously increased in both glioma cell lines and glioma tissues. Second, to evaluate the effect of NUP37 on the malignant biological behavior of glioma cells, siRNA was used to interfere with the expression of NUP37 in A172 cell. The knockdown efficiency of siRNA was shown in Figure 8C, we used siRNA#2 for subsequent experiments. Third, wound healing experiments showed that the wound healing ability of the experimental group was significantly reduced compared with the control group (Figure 9A). Fourth, colony formation analysis showed that NUP37 siRNA was able to suppress the colony formation ability of A172 cells (Figure 9B). Finally, immunofluorescence results showed that reduction of NUP37 expression was able to inhibit A172 cell proliferation (Figure 9C).

**FIGURE 10:**
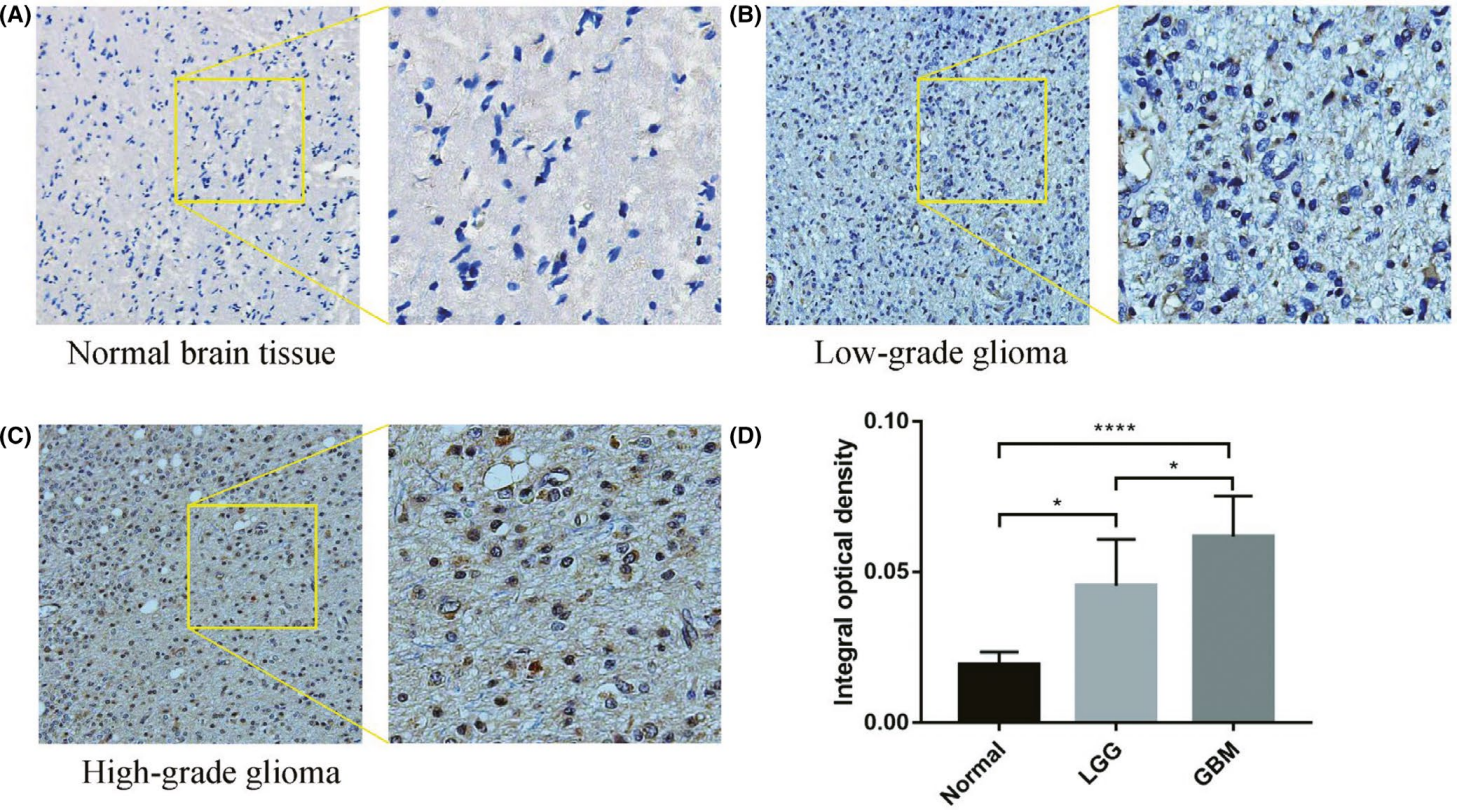
The staining of NUP37 in normal brain (A); lightly positive staining of NUP37 in low‐grade glioma (B); strongly positive staining of NUP37 in high‐grade glioma (C); the expression of NUP37 in each group was statistically analyzed (D). Original magnification, A–C = ×200

All the data were based on the study of NUP37 mRNA level, so we further verified the change of NUP37 protein level in glioma. First, we validated the protein expression levels of NUP37 in glioma using HPA database. As shown in Figure S4, NUP37 expression was slightly increased in gliomas of different grades compared to normal brain tissue. Finally, immunohistochemistry was also done to analyze the expression levels of NUP37 protein in normal brain tissue, low‐grade glioma, and high‐grade glioma using collected clinical samples (4 normal brain samples, 7 low‐grade glioma samples, and 19 high‐grade glioma samples). As shown in Figure 10, the protein expression level of NUP37 was obviously increased in glioma tissues.

## 4 DISCUSSION

In the pathological process of glioma, some cancer‐related genes have been found and elaborated, but there are few reports about the relationship between NUP37 and the prognosis and clinical characteristics of glioma. Previous studies have shown that the expression level of NUP37 as an oncogene was significantly increased in tumor tissues, but it is not known in glioma.^24^ In this study, we systematically explored the expression changes of NUP37 between gliomas and normal tissues through several dimensions of thousands of tissue samples. First, as shown in Figure 1, the expression level of NUP37 in glioma tissues was significantly higher than that in normal brain tissues in both in GEPIA and GEO databases. Second, RT‐PCR technology was further used to detect the expression level of NUP37 in both clinical glioma samples and glioma cell lines. Compared with the control group, the expression level of NUP37 was significantly increased, which was consistent with the data from the public database. Finally, as above results were at the mRNA level, we further used immunohistochemistry to detect the protein expression of NUP37, and found that the protein expression level of NUP37 in glioma was also higher than that in the normal brain tissue. Collectively, our analysis gathered thousands of glioma samples and emphasized that the expression level of NUP37 increased in the pathological progression of glioma, which laid the foundation for our further analysis.

Oncogenes are often closely related to the prognosis and clinical characteristics of patients with cancer, so we want to further reveal the relationship between NUP37 and the clinical and molecular characteristics of gliomas. First, the high expression of NUP37 was closely related to a variety of prognosis‐related clinical characteristics.

In particular, the expression level of NUP37 was positively correlated with the grade of glioma, which also supports that NUP37 is a malignant molecule. Second, IDH mutations and 1p19q codeletion are protective factors for the prognosis of glioma patients,^25^ but the expression of NUP37 is negatively correlated with them. Finally, we next compare the relationship between NUP37 and known biomarkers in gliomas. Through correlation analysis, we found that NUP37 was positively correlated with most of the known biomarkers.^26^ For example, EGFR and STAT3 were both highly expressed in gliomas and negatively correlated with the overall survival of gliomas.^27^, ^28^, ^29^ In addition, as the expression of NUP37 was highly positively correlated with the genes encoding PD‐L1 (CD274) and PD‐L2 (PDCD1LG2), it is more importantly that PD‐L1 and PD‐L2 are an immune checkpoint inhibitor in gliomas. Their increased expression level can lead to the immune escape of glioma cells to T cell.^30^ Therefore, the malignant characteristics of NUP37 may be partly due to the coordination between PD‐L1 and PD‐L2, which play a role by regulating the activity of T cells.

As a typical feature of oncogene, it has an impact on the prognosis of cancer patients and previous reports also confirmed that the increased expression of NUP37 can significantly reduce the survival time of lung cancer patients.^19^ In this study, we also revealed the association between NUP37 expression and survival of glioma patients by a variety of data types to verify each other (CGGA RNA‐seq, CGGA microarray, and TCGA RNA‐seq). Encouragingly, compared with the high expression group of NUP37, the low expression group of NUP37 is closely related to the better prognosis of glioma patients. This conclusion was further supported by multivariate analysis and meta‐analysis that high expression of NUP37 can be used as an independent risk factor to reduce the overall survival time of glioma patients. However, in glioblastoma, only the CGGA database showed that the high expression of NUP37 leads to the decrease of the overall survival time of glioma patients. It is well known that glioblastoma is the main pathological type leading to poor prognosis of glioma.^31^ Therefore, in order to make up for the difference between the 3 databases, we collected 23 cases of glioblastoma to drew the survival curve, and found that the high expression of NUP37 in glioblastoma had only weak statistical significance on the prognosis of gliomas. Finally, knockdown of NUP37 showed that the proliferation and migration ability of A172 glioma cell line were significantly decreased and previous articles also support the view that NUP37 is an oncogene.^18^, ^24^ Collectively, the core point of the above discussion is that NUP37 may be a potential target for predicting the prognosis of glioma patients.

Although this study has identified the potential diagnostic value of NUP37 in the prognosis of glioma, the regulatory mechanism of NUP37 remains to be explored. Therefore, GSEA analysis is used to indirectly reveal the effect of NUP37 on cellular signaling pathways. The results showed that NUP37 can activate a variety of carcinogenic signaling pathways, which have been reported in the pathological progress of glioma. For example, MiR‐188 plays an important regulatory role in the pathological process of glioma, affecting the signal of cell cycle, which can inhibit the biological behavior of glioma cells.^32^ DNA replication requires the interaction of multiple proteins and enzymes and microRNA‐31 inhibits glioma cell proliferation by inhibition of DNA replication.^33^ Mismatch repair has an important relationship with the occurrence of many kinds of tumors and has an important impact on the resistance of chemotherapeutic drugs.^34^, ^35^, ^36^ Therefore, through the above discussion, we have enough reasons to believe that the carcinogenic effect of NUP37 in glioma was realized via cell cycle, mismatch repair, and DNA replication signaling pathway.

It is of great value to reveal the mechanism of oncogenes in tumors for the formulation of clinical treatment strategies. Recognizing that some tumors contain abnormal expression of coding proteins can be targeted for the development of small molecule inhibitors. Based on this rationale, we employed CMap analysis to obtain small molecule drugs with inhibitory effects on NUP37, and finally we obtained four drugs (cycloheximide, riboflavin, levamisole, and emetine) according to the cut‐off criterion (*p* < 0.01 and enrichment < −0.7). The formulation of this cut‐off criterion was based on the rationale of CMap analysis and the enrichment index was set up from −1 to 1, which indicates the strength of inhibition of the target gene by the drug.^37^ In other words, the closer the enrichment index of a certain drug was to −1 and the more obvious was the inhibitory effect on the target gene. Our small molecule drugs have been obtained to have inhibitory effect on cancer in previous studies. For example, Wang et al. found that cycloheximide promoted NIM811‐induced paraptosis, caused paraptotic cell death of glioblastoma cells, and inhibited the growth of GBM.^38^ Koppany Visnyei et al. confirmed that emetine inhibited the proliferation of glioma cells.^39^ Levamisole minimizes drug side effects and inhibits the activity of ovarian cancer cells.^40^ Riboflavin has a blue light activation effect, which produce reactive oxygen species (ROS) to inhibit the activity of cancer cells.^41^ Some drugs have new therapeutic effects that allow them play an unexpected role in the treatment of certain diseases, which accelerates the process of drug development. For example, aspirin is widely used as an analgesic and anti‐inflammatory drug, and it was later found to have a preventive effect on colorectal cancer. Therefore, we suggest that the four small molecule drugs found in the present study have a certain potential therapeutic effect on glioma.

It is worth noting that some of the deficiencies in this study are unavoidable. As a retrospective study, all clinical features and treatment options of patients should be included in the analysis as far as possible. However, most of the data sources of this analysis are from public databases, so the detailed diagnosis and treatment plan are not included in the analysis, which is also an inherent defect of public databases. Especially in TCGA database, the IDH mutation and 1p19q codeletion, which have important prognostic value, were missing. For this important information based on the pathological grading of glioma in 2016, the public database has no ability to correct this timeliness due to its own factors. However, multi‐database analyses have the advantages of multi‐center, multi‐ethnic, and large samples. Second, the imbalance between the number of samples of normal brain tissue and glioma tissue is improved after the study. Consequently, in order to make up for these deficiencies, we used the traditional experimental methods to verify the carcinogenic effect of NUP37 in glioma.

## 5 CONCLUSION

It is the first time to explain that NUP37 as an oncogene can be used as an independent risk factor leading to poor prognosis of glioma and has clinical diagnostic value, especially in grade III glioma. We partially revealed the mechanism of NUP37 in glioma and provided a potential diagnostic and therapeutic target.

## 6 AUTHOR CONTRIBUTIONS

ZDL and HBW conceived and designed the experiments. YLJ contributed reagents/materials/analysis tools, reviewed drafts of the paper. JLW, YBW, LB and BFL collected data. XYL, BZ, ZSR and WZ analyzed the data, prepared figures and/or tables. YZG and WWD contributed reagents/materials/analysis tools, authored or reviewed drafts of the paper, approved the final draft.

## CONFLICT OF INTEREST

The authors declare there are no competing interest.

## ETHICS STATEMENT

The study protocol was approved by the Ethics Committee of the Henan Provincial People's Hospital (Zhengzhou, China). The use of patient samples conformed to the declaration of Helsinki. All patients provided informed written consent.

## Supporting information

Figure S1

Figure S2

Figure S3

Figure S4

Table S1

Table S2

Table S3

## Data Availability

Data on the results of this study are available from the corresponding author.
