# Supplementary figures and images for: Significantly high expression of NUP37 leads to poor prognosis of glioma patients by promoting the proliferation of glioma cells
Source: Cancer Med. 2021 Jul 15;10(15):5218–34. doi: 10.1002/cam4.3954 (PMC8335818; doi:10.1002/cam4.3954)

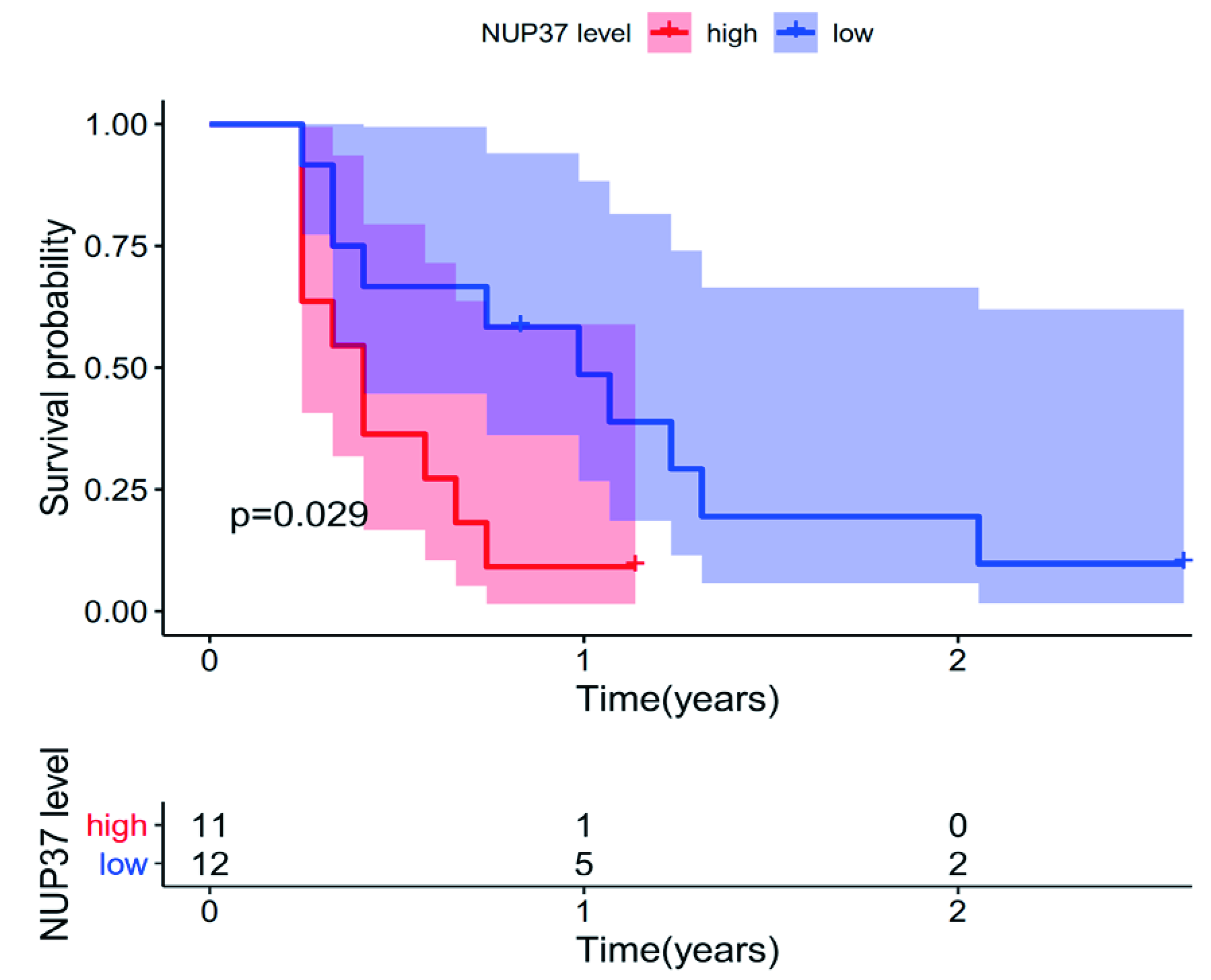

Supplement: Supplementary file 1 — Figure S1 [file CAM4-10-5218-s003.tif]

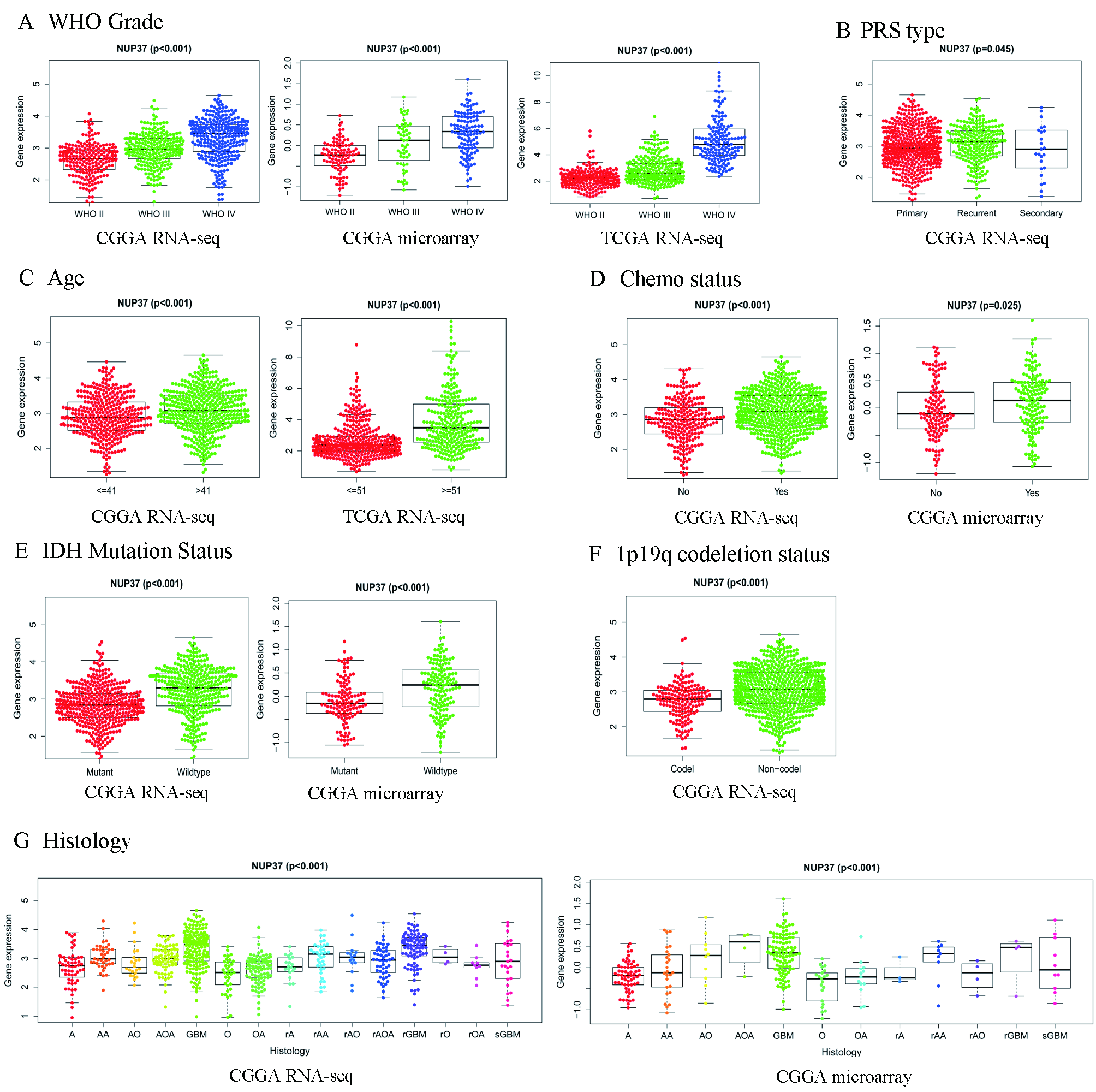

Supplement: Supplementary file 2 — Figure S2 [file CAM4-10-5218-s002.tif]

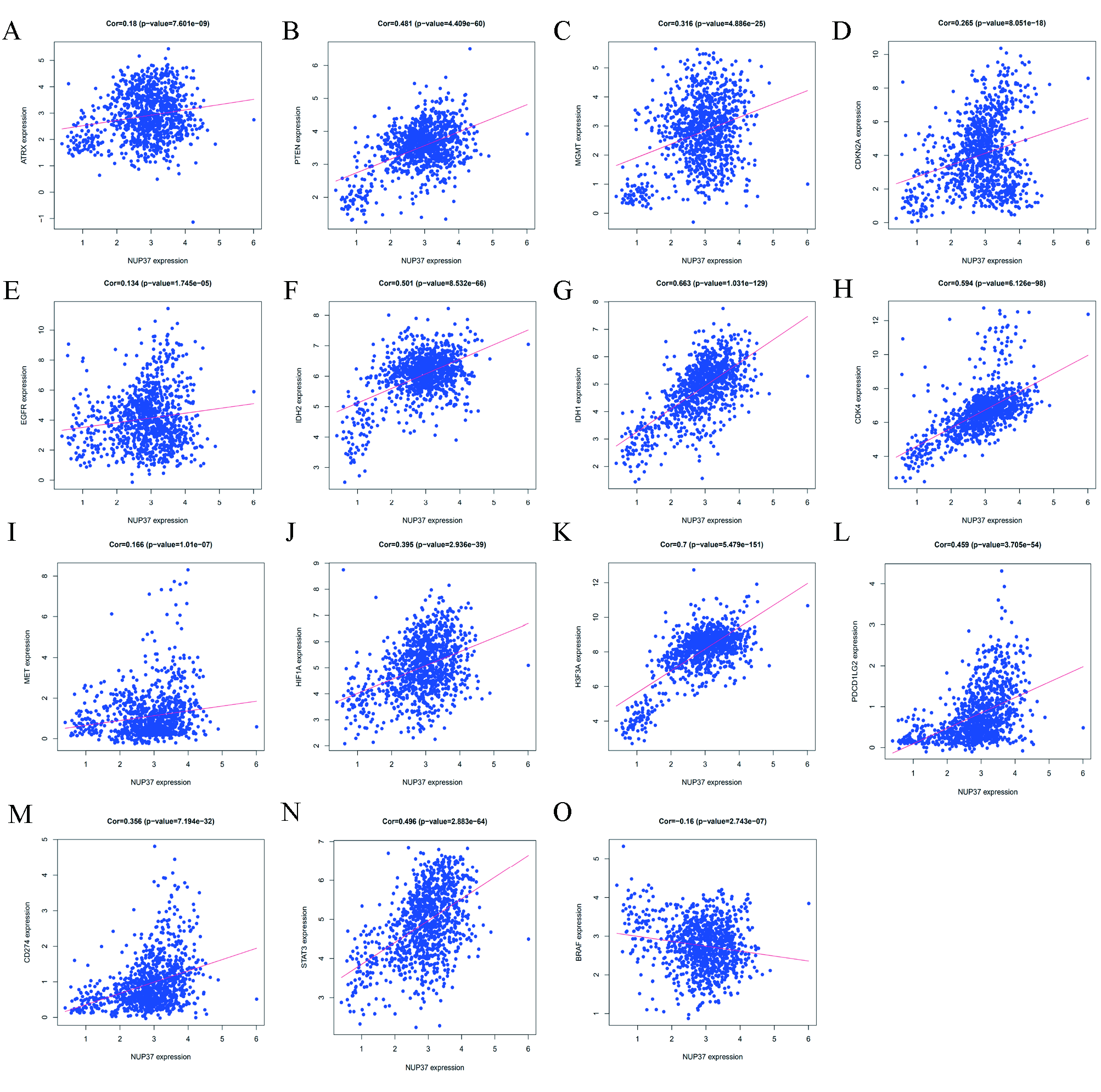

Supplement: Supplementary file 3 — Figure S3 [file CAM4-10-5218-s007.tif]

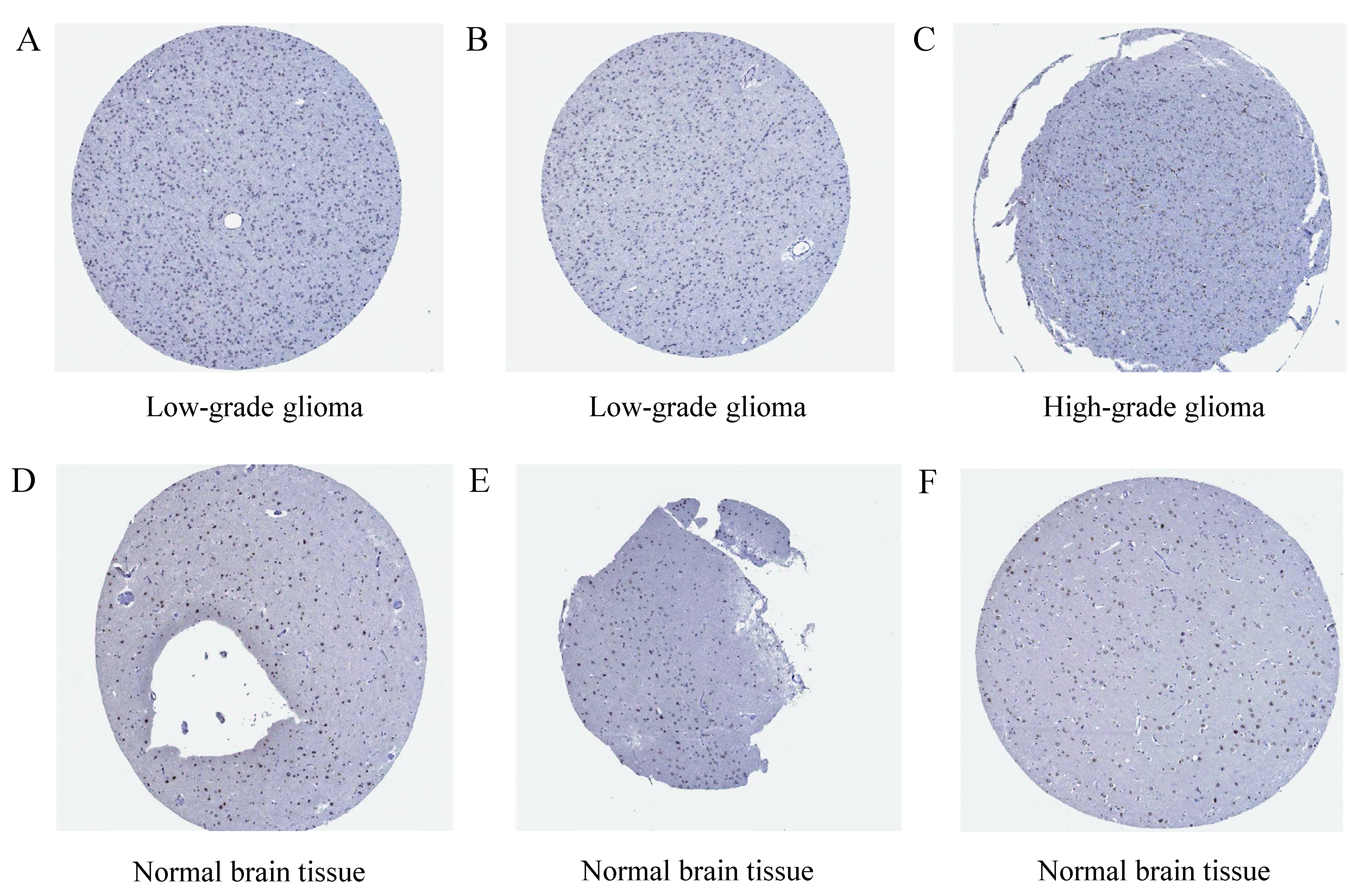

Supplement: Supplementary file 4 — Figure S4 [file CAM4-10-5218-s001.tif]
