# Supplementary material for: Significantly high expression of NUP37 leads to poor prognosis of glioma patients by promoting the proliferation of glioma cells
Source: Cancer Med. 2021 Jul 15;10(15):5218–34. doi: 10.1002/cam4.3954 (PMC8335818; doi:10.1002/cam4.3954)
Supplement: Supplementary file 6 — Table S2 [file CAM4-10-5218-s005.docx]

Table S2. Characteristics of patients with glioma based on CGGA microarray.

| Characteristics |  | Number of cases | Percentages (%) |
| --- | --- | --- | --- |
| Gender | Male | 153 | 57.09 |
|  | Female | 115 | 42.91 |
| Age | ≤42 | 138 | 51.49 |
|  | >42 | 130 | 48.51 |
| Grade | WHO II | 100 | 37.31 |
|  | WHO III | 52 | 19.41 |
|  | WHO IV | 116 | 43.28 |
| PRS_type | Primary | 238 | 88.81 |
|  | Recurrent | 20 | 7.46 |
|  | Secondary | 10 | 3.73 |
| Radio_status | Yes | 240 | 89.55 |
|  | No | 28 | 10.45 |
| Chemo_status | Yes | 145 | 54.10 |
|  | No | 123 | 45.90 |
| Histology | Astrocytoma | 63 | 23.51 |
|  | Anaplastic astrocytoma | 25 | 9.33 |
|  | Anaplastic Oligodendroglioma | 10 | 3.73 |
|  | Anaplastic oligoastrocytoma | 4 | 1.49 |
|  | Glioblastoma | 102 | 38.06 |
|  | Oligodendroglioma | 21 | 7.84 |
|  | oligoastrocytoma | 13 | 4.85 |
|  | relapse astrocytoma | 3 | 1.12 |
|  | relapse Anaplastic astrocytoma | 9 | 3.36 |
|  | relapse Anaplastic Oligodendroglioma | 4 | 1.49 |
|  | relapse oligoastrocytoma | 4 | 1.49 |
|  | Secondary relapse Oligodendroglioma | 10 | 3.73 |
| IDH_mutation_status | Mutant | 121 | 45.15 |
|  | Wildtype | 147 | 54.85 |
