# Supplementary material for: Significantly high expression of NUP37 leads to poor prognosis of glioma patients by promoting the proliferation of glioma cells
Source: Cancer Med. 2021 Jul 15;10(15):5218–34. doi: 10.1002/cam4.3954 (PMC8335818; doi:10.1002/cam4.3954)
Supplement: Supplementary file 7 — Table S3 [file CAM4-10-5218-s004.docx]

Table S3. Characteristics of patients with glioma based on TCGA

| Characteristics |  | Number of cases | Percentages (%) |
| --- | --- | --- | --- |
| Gender | Male | 377 | 57.73 |
|  | Female | 276 | 42.27 |
| Age | ≤51 | 394 | 60.34 |
|  | >51 | 259 | 39.66 |
| Grade | WHO II | 238 | 36.45 |
|  | WHO III | 256 | 39.20 |
|  | WHO IV | 159 | 24.35 |
